# T3 Sexual Satisfaction and Association with Psychosocial Outcomes Among Burn Survivors

**DOI:** 10.1093/jbcr/irad045.003

**Published:** 2023-05-15

**Authors:** Liam D Cato, Lauren J Shepler, Kara McMullen, Kimberly Roaten, Lewis E Kazis, Colleen M Ryan, Jeffrey C Schneider

**Affiliations:** Boston Children's Hospital / Harvard Medical School, Brookline, Massachusetts; Spaulding Rehabilitation Hospital, Harvard Medical School, Boston, Massachusetts; University of Washington, Seattle, Washington; UT Southwestern Medical Center/Parkland Health, Dallas, Texas; Boston University School of Public Health, Rehabilitation Outcomes Center at Spaulding, Boston, Massachusetts; Massachusetts General Hospital, Boston, Massachusetts; Spaulding Rehabilitation Hospital/Harvard Medical School/Rehabilitation Outcomes Center at Spaulding, Boston, Massachusetts; Boston Children's Hospital / Harvard Medical School, Brookline, Massachusetts; Spaulding Rehabilitation Hospital, Harvard Medical School, Boston, Massachusetts; University of Washington, Seattle, Washington; UT Southwestern Medical Center/Parkland Health, Dallas, Texas; Boston University School of Public Health, Rehabilitation Outcomes Center at Spaulding, Boston, Massachusetts; Massachusetts General Hospital, Boston, Massachusetts; Spaulding Rehabilitation Hospital/Harvard Medical School/Rehabilitation Outcomes Center at Spaulding, Boston, Massachusetts; Boston Children's Hospital / Harvard Medical School, Brookline, Massachusetts; Spaulding Rehabilitation Hospital, Harvard Medical School, Boston, Massachusetts; University of Washington, Seattle, Washington; UT Southwestern Medical Center/Parkland Health, Dallas, Texas; Boston University School of Public Health, Rehabilitation Outcomes Center at Spaulding, Boston, Massachusetts; Massachusetts General Hospital, Boston, Massachusetts; Spaulding Rehabilitation Hospital/Harvard Medical School/Rehabilitation Outcomes Center at Spaulding, Boston, Massachusetts; Boston Children's Hospital / Harvard Medical School, Brookline, Massachusetts; Spaulding Rehabilitation Hospital, Harvard Medical School, Boston, Massachusetts; University of Washington, Seattle, Washington; UT Southwestern Medical Center/Parkland Health, Dallas, Texas; Boston University School of Public Health, Rehabilitation Outcomes Center at Spaulding, Boston, Massachusetts; Massachusetts General Hospital, Boston, Massachusetts; Spaulding Rehabilitation Hospital/Harvard Medical School/Rehabilitation Outcomes Center at Spaulding, Boston, Massachusetts; Boston Children's Hospital / Harvard Medical School, Brookline, Massachusetts; Spaulding Rehabilitation Hospital, Harvard Medical School, Boston, Massachusetts; University of Washington, Seattle, Washington; UT Southwestern Medical Center/Parkland Health, Dallas, Texas; Boston University School of Public Health, Rehabilitation Outcomes Center at Spaulding, Boston, Massachusetts; Massachusetts General Hospital, Boston, Massachusetts; Spaulding Rehabilitation Hospital/Harvard Medical School/Rehabilitation Outcomes Center at Spaulding, Boston, Massachusetts; Boston Children's Hospital / Harvard Medical School, Brookline, Massachusetts; Spaulding Rehabilitation Hospital, Harvard Medical School, Boston, Massachusetts; University of Washington, Seattle, Washington; UT Southwestern Medical Center/Parkland Health, Dallas, Texas; Boston University School of Public Health, Rehabilitation Outcomes Center at Spaulding, Boston, Massachusetts; Massachusetts General Hospital, Boston, Massachusetts; Spaulding Rehabilitation Hospital/Harvard Medical School/Rehabilitation Outcomes Center at Spaulding, Boston, Massachusetts; Boston Children's Hospital / Harvard Medical School, Brookline, Massachusetts; Spaulding Rehabilitation Hospital, Harvard Medical School, Boston, Massachusetts; University of Washington, Seattle, Washington; UT Southwestern Medical Center/Parkland Health, Dallas, Texas; Boston University School of Public Health, Rehabilitation Outcomes Center at Spaulding, Boston, Massachusetts; Massachusetts General Hospital, Boston, Massachusetts; Spaulding Rehabilitation Hospital/Harvard Medical School/Rehabilitation Outcomes Center at Spaulding, Boston, Massachusetts

## Abstract

**Introduction:**

Sexual satisfaction is often unaddressed in clinical care despite it being an important aspect of healing and quality of life for burn survivors. Previous literature has focused predominantly on physical function rather than perceived satisfaction. This study aimed to determine the associations of sexual satisfaction with psychosocial outcomes and assess sexual satisfaction recovery over time after burn injury.

**Methods:**

Patients consenting to provide data to the Burn Model System (BMS) National Database injured after 2015, aged greater than 18 years at the time of burn injury, and alive at discharge were included in the analysis. Sexual satisfaction was collected at six months and then every year for five years post-injury using two PROMIS sexual satisfaction items, a composite T-score was derived, where a higher score indicated higher sexual satisfaction. Analyses included sexual satisfaction scores as outcomes with mixed models that included repeated measures controlling for correlated error of the outcomes and covariates that included demographics. Psychosocial measures analyzed included: PROMIS-29 Anxiety, Depression, Fatigue and Sleep scores; VR-12 mental component score (MCS) and physical component score (PCS); and the Post-Traumatic Growth Inventory (PTGI) total score.

**Results:**

The analysis identified 561 individuals with sexual satisfaction data for at least one time point. The population demographics included a median age of 46 years (IQR 34, 59), reported sex (female = 171 (30.5%)), and median TBSA burned 10% (IQR 3, 26). Increasing age (p < 0.0001) and marital status with those ‘Divorced’ (p=0.004) or ‘Single’ (p=0.0003) were associated with lower sexual satisfaction. There was no association with TBSA burn size. Within psychosocial metrics, we found that the CIQ Social Integration score (p < 0.0001), VR-12 MCS (p=0.008), PTGI Total Score (p=0.01), and PROMIS 29 Sleep Disturbance score (p=0.0004) were also significantly associated. Change in scores were associated with the VR-12 MSC (p=0.03), where an increased MCS score predicted improved sexual satisfaction by final follow-up.

**Conclusions:**

A significant association was found between sexual satisfaction and social integration, mental health, and post-traumatic growth.

**Applicability of Research to Practice:**

These findings suggest that interventions to improve the sexual satisfaction of burn survivors should focus on the importance of social integration, mental health, and sleep quality.

